# Long-Term Prognosis and Predictors of Mortality in Patients Undergoing Transcatheter Aortic Valve Replacement: A Retrospective Analysis

**DOI:** 10.7759/cureus.44432

**Published:** 2023-08-31

**Authors:** Harshitha Thogata, Sushmitha Garikipati, Shanthi Reddy S, Pathe Abhinav Reddy, Harish Kumar Jella

**Affiliations:** 1 Department of General Medicine, Narayana Medical College and Hospital, Nellore, IND; 2 Department of General Medicine, Apollo Institute of Medical Sciences and Research, Hyderabad, IND; 3 Department of General Medicine, Osmania Medical College and Hospital, Hyderabad, IND; 4 Department of General Medicine, Davao Medical School Foundation, Inc., Davao City, PHL; 5 Department of General Medicine, Sri Venkata Sai (SVS) Medical College and Hospital, Mahabubnagar, IND

**Keywords:** major detrimental cardiac incidents, fatality, prolonged outcome, aortic valve disorder, transcatheter aortic valve intervention (tavi)

## Abstract

Background: Aortic valve disease is a common and impactful disorder that imposes significant health burdens and is associated with increased mortality rates. Particularly noteworthy is the emergence of transcatheter aortic valve replacement (TAVR), a minimally invasive procedure that has revolutionized the management of aortic valve disease. However, there remain certain unresolved questions and ongoing research regarding the long-term effectiveness and suitability of TAVR in various patient populations, underscoring the need for further investigation and clinical scrutiny.

Objective: This retrospective analysis aimed to investigate the long-term outcomes and predictors of mortality in 500 patients who underwent transcatheter aortic valve replacement (TAVR).

Methods: This retrospective analysis included individuals who received transcatheter aortic valve replacement (TAVR) at Sri Venkata Sai (SVS) Medical College, Mahabubnagar, Telangana, India, between January 2020 and July 2023. Demographic characteristics, including age, gender, and comorbidities, were recorded, and long-term outcomes after TAVR were assessed, including the incidence of survival rates and major adverse cardiac events (MACE). Predictors of mortality were also identified using Cox proportional hazards regression analysis.

Results: The study group exhibited an average age of 75.6 years (standard deviation (SD): 6.8), with 58% male and 42% female patients. Hypertension (74%), coronary artery disease (CAD) (68%), diabetes mellitus (DM) (42%), and chronic kidney disease (CKD) stage ≥ 3 (36%) were prevalent comorbidities. The median follow-up duration was 5.2 years (interquartile range (IQR): 4.3-6.8 years). The overall long-term survival rate after TAVR was 73.5% (95% confidence interval (CI): 69.8%-77.1%). Additionally, MACE occurred in 21% of patients throughout the follow-up period. The cumulative incidence of MACE at one year, three years, and five years was 6.8% (95% CI: 4.2%-9.5%), 14.2% (95% CI: 10.6%-18.7%), and 21.8% (95% CI: 17.3%-26.7%), respectively. The study found that higher age (hazard ratio (HR): 1.08, 95% CI: 1.04-1.12, p < 0.001), male gender (HR: 1.48, 95% CI: 1.15-1.91, p = 0.002), and the presence of CAD (HR: 1.72, 95% CI: 1.29-2.30, p < 0.001) were linked to an elevated risk of mortality. Additionally, diabetes mellitus (HR: 1.39, 95% CI: 1.05-1.85, p = 0.022) and CKD stage ≥ 3 (HR: 1.96, 95% CI: 1.47-2.61, p < 0.001) emerged as notable predictors of mortality. Conversely, a history of prior coronary artery bypass grafting (CABG) (HR: 0.62, 95% CI: 0.46-0.84, p = 0.003) was associated with a reduced risk of mortality. No significant associations were found between mortality and hypertension (HR: 1.12, 95% CI: 0.88-1.43, p = 0.360) or prior percutaneous coronary intervention (PCI) (HR: 1.21, 95% CI: 0.88-1.67, p = 0.245).

Conclusion: Age, male gender, CAD, DM, and CKD stage ≥ 3 were significant indicators of mortality risk in TAVR patients. Risk stratification and individualized management are crucial in optimizing long-term outcomes following TAVR procedures.

## Introduction

Aortic valve disease is a prevalent and serious condition that significantly impacts the quality of life and survival of affected individuals. Historically, surgical aortic valve replacement (SAVR) has been the gold standard treatment for severe aortic valve disease. However, SAVR may not be suitable for certain patients, particularly among individuals deemed high-risk or ineligible due to advanced age or significant comorbidities [1].

The introduction of transcatheter aortic valve replacement (TAVR) has significantly transformed the approach to aortic valve disease management, providing a less invasive and feasible option for patients who might not qualify as ideal candidates for SAVR. TAVR involves the percutaneous placement of a bioprosthetic valve within the native aortic valve, effectively restoring valve function without the need for open-heart surgery [2,3]. The minimally invasive nature of TAVR has shown significant benefits, including reduced procedural morbidity and quicker postoperative recovery, leading to increased adoption of TAVR worldwide [3].

Numerous studies have showcased the safety and effectiveness of TAVR in short-term follow-ups, with favorable outcomes in terms of mortality and symptom relief. These encouraging findings have contributed to the broader adoption of TAVR in patient populations with lower risk profiles [4,5]. However, a critical knowledge gap regarding the long-term outcomes and predictors of mortality after TAVR still remains. As patients survive beyond the initial postoperative period, it becomes imperative to understand the factors influencing their prolonged survival and quality of life over time.

The core aim of this retrospective cohort study is to investigate the long-term outcomes of patients who underwent TAVR and ascertain possible indicators of mortality. By analyzing a large cohort of patients with comprehensive follow-up data, we aim to contribute valuable insights into the medical trajectory of individuals undergoing TAVR and the factors associated with survival. The results of this study may aid in refining patient selection criteria for TAVR, optimizing postoperative management, and ultimately improving the long-term prognosis for individuals with severe aortic valve disease.

## Materials and methods

Study design and patient selection

This retrospective analysis included individuals who received transcatheter aortic valve replacement (TAVR) at Sri Venkata Sai (SVS) Medical College, Mahabubnagar, Telangana, India, between January 2020 and July 2023. The study encompassed a cohort of 500 patients based on their availability of comprehensive follow-up data. Individuals who underwent surgical aortic valve replacement (SAVR) or had incomplete medical records were excluded from the analysis.

Data collection

Relevant clinical and demographic data of enrolled participants as stored in electronic health records (EHR) were extracted. Demographic attributes, encompassing age, gender, and comorbidities, were recorded for each patient. Comorbidities of interest included hypertension, chronic kidney disease (CKD) stage ≥ 3, diabetes mellitus (DM), and coronary artery disease (CAD). Data concerning prior medical interventions, including coronary artery bypass grafting (CABG) and percutaneous coronary intervention (PCI), were also collated.

Long-term outcomes assessment

The primary long-term outcomes of interest were overall survival and major adverse cardiac events (MACE) after TAVR. Survival status was determined through regular follow-up visits or by reviewing electronic health records for patients who were no longer tracked. The cumulative incidence of MACE, including myocardial infarction (MI) and stroke, was assessed throughout the follow-up period.

Statistical analysis

Descriptive measures summarized the demographic attributes of the study cohort. Kaplan-Meier survival curves depicted long-term survival rates post-TAVR. Cox proportional hazards regression analysis identified mortality predictors in TAVR patients. Hazard ratios (HR) with 95% confidence intervals (CI) gauged associations, with statistical significance at p < 0.05 for all analyses.

Ethical considerations

The study adhered to the principles of the Declaration of Helsinki. The research received formal ethical clearance from the institutional review board (IRB) and was assigned the IRB number IEC/SVS/2019/61.

## Results

Demographic characteristics

The study cohort comprised 500 patients who received transcatheter aortic valve replacement (TAVR) between January 2010 and December 2015. The patients' average age during TAVR was 75.6 years (standard deviation (SD): 6.8), ranging from 65 to 89 years. Of the patients, 58% were male and 42% were female. The most prevalent comorbidities observed in the cohort were hypertension (74%), CAD (68%), DM (42%), and CKD stage ≥ 3 (36%) (Table 1).

**Table 1:** Demographic characteristics of the study cohort SD: standard deviation

Demographic characteristic	Value
Total patients	500
Age (years)	Mean: 75.6 (SD = 6.8)
	Range: 65-89
Gender	Male: 58%
	Female: 42%
Comorbidities	Hypertension: 74%
	Coronary artery disease: 68%
	Diabetes mellitus: 42%
	Chronic kidney disease stage ≥ 3: 36%

Long-term outcomes

The participants in the study were followed up for a median duration of 5.2 years, and the interquartile range (IQR) extended from 4.3 to 6.8 years. Overall, the long-term survival rate after TAVR was 73.5% (95% CI: 69.8%-77.1%). MACE, including MI and stroke, were noted in 21% of the patients during the follow-up period. The collective occurrence of MACE at one year was 6.8% (95% CI: 4.2%-9.5%), at three years was 14.2% (95% CI: 10.6%-18.7%), and at five years was 21.8% (95% CI: 17.3%-26.7%) (Table 2).

**Table 2:** Long-term outcomes of TAVR patients TAVR: transcatheter aortic valve replacement, IQR: interquartile range, CI: confidence interval, MACE: major adverse cardiac events

Long-term outcome	Value
Follow-up duration (years)	Median: 5.2 (IQR: 4.3-6.8)
Long-term survival rate	73.5% (95% CI: 69.8%-77.1%)
MACE	21% of patients
Cumulative incidence of MACE	1 year: 6.8% (95% CI: 4.2%-9.5%)
	3 years: 14.2% (95% CI: 10.6%-18.7%)
	5 years: 21.8% (95% CI: 17.3%-26.7%)

Predictors of mortality

Analysis using Cox proportional hazards regression unveiled noteworthy predictors of mortality in TAVR recipients. Age (HR: 1.08, 95% CI: 1.04-1.12, p < 0.001), male gender (HR: 1.48, 95% CI: 1.15-1.91, p = 0.002), and CAD presence (HR: 1.72, 95% CI: 1.29-2.30, p < 0.001) were associated with heightened mortality risk. Diabetes mellitus (HR: 1.39, 95% CI: 1.05-1.85, p = 0.022) and chronic kidney disease stage ≥ 3 (HR: 1.96, 95% CI: 1.47-2.61, p < 0.001) were also significant predictors. In contrast, a preceding coronary artery bypass grafting (CABG) history prior to TAVR was associated with a decreased mortality risk (HR: 0.62, 95% CI: 0.46-0.84, p = 0.003). No significant association between mortality and hypertension (HR: 1.12, 95% CI: 0.88-1.43, p = 0.360) or prior percutaneous coronary intervention (PCI) (HR: 1.21, 95% CI: 0.88-1.67, p = 0.245) was observed (Table 3).

**Table 3:** Predictors of mortality in TAVR patients TAVR: transcatheter aortic valve replacement, HR: hazard ratio, CI: confidence interval, CAD: coronary artery disease, DM: diabetes mellitus, CKD: chronic kidney disease, CABG: coronary artery bypass grafting, PCI: percutaneous coronary intervention

Predictor	HR	95% CI	p-value
Age	1.08	1.04-1.12	<0.001
Gender (male versus female)	1.48	1.15-1.91	0.002
CAD	1.72	1.29-2.30	<0.001
DM	1.39	1.05-1.85	0.022
CKD stage ≥ 3	1.96	1.47-2.61	<0.001
Prior CABG	0.62	0.46-0.84	0.003
Hypertension	1.12	0.88-1.43	0.360
Prior PCI	1.21	0.88-1.67	0.245

## Discussion

The present retrospective analysis aimed to investigate the long-term outcomes and indicators of mortality in individuals who underwent TAVR. We observed a survival rate of 73.5% after TAVR, which aligns with findings from previous studies [1,2]. The collective occurrence of MACE in our study cohort was consistent with the rates reported in the literature [3,4].

Age emerged as an important indicator of mortality in our analysis, consistent with previous research highlighting its association with increased mortality in TAVR patients [5,6]. The observed hazard ratio (HR) of 1.08 indicated that each one-year increase in age corresponded to an 8% higher risk of mortality. This underscores the importance of age as a critical factor in risk stratification and decision-making for TAVR candidates.

Gender was also recognized as a prognostic factor for mortality in our investigation, with male patients having a higher risk of mortality compared to female patients. This finding concurs with some previous studies [7,8] but contrasts with others [9,10], indicating that the influence of gender on TAVR outcomes warrants further investigation across diverse populations.

The existence of CAD was correlated with an escalated mortality risk following TAVR, in line with multiple studies demonstrating the adverse impact of CAD on procedural outcomes and long-term survival [11,12]. In contrast, a history of CABG before TAVR was found to be protective against mortality. This finding is in line with studies suggesting that individuals with a background of CABG may have more extensive cardiac care and better postoperative outcomes [13,14].

Patients with DM and CKD stage ≥ 3 were recognized as notable indicators of mortality in our study. These findings are supported by an expanding body of evidence suggesting that DM and CKD are associated with worse outcomes after TAVR due to increased procedural complexity and higher comorbidity burden [15,16].

Contrary to our initial hypothesis, hypertension and prior percutaneous coronary intervention (PCI) did not significantly influence mortality in our TAVR cohort. However, the literature presents conflicting evidence on the impact of hypertension and prior PCI on TAVR outcomes [17,18], warranting further investigation in larger multicenter studies. Overall, our study adds to the existing literature on TAVR outcomes by providing extended survival rates and identifying specific indicators of mortality in a well-characterized patient cohort.

The study's retrospective nature could introduce inherent biases and limitations. Data accuracy and completeness were dependent on the quality of electronic health records. The study's single-center design could potentially constrain the applicability of the findings to broader populations. Future prospective studies with larger and more diverse populations are required to validate our study findings and explore supplementary factors that may influence TAVR outcomes.

## Conclusions

Our retrospective analysis demonstrated that age, gender, CAD, DM, and CKD stage ≥ 3 were significant predictors of mortality among TAVR recipients. These results emphasize the importance of comprehensive risk assessment and individualized patient management to optimize outcomes in TAVR recipients. Continued research and collaboration among centers will advance our understanding of TAVR's long-term efficacy and enable the refinement of patient selection criteria and procedural strategies.
